# Interleukin-22 Suppresses the Growth of A498 Renal Cell Carcinoma Cells via Regulation of STAT1 Pathway

**DOI:** 10.1371/journal.pone.0020382

**Published:** 2011-05-23

**Authors:** Fengbo Zhang, Donghao Shang, Yuhai Zhang, Ye Tian

**Affiliations:** Department of Urology Surgery, Beijing Friendship Hospital, Capital Medical University, Beijing, China; Emory University, United States of America

## Abstract

**Background:**

Renal cell carcinoma (RCC) is one of the most common kidney cancers and is highly resistant to chemotherapy. Accumulating evidence suggests that interleukin-22 (IL-22) may mediate host defense against varietal pathogens as a proinflammatory and anti-inflammatory cytokine. The purpose of this study is to assess the inhibitory effects of IL-22 on human RCC cell line A498 and to investigate the possible mechanisms underlying the anti-tumor effects of this cytokine.

**Methodology:**

A498 cells, a RCC cell line, were used to assess the inhibitory growth effects of IL-22 using the MTT assay and flow cytometric analysis in vitro. BALB/C nude mice bearing A498 cell xenografts were used to examine the antitumor efficacy of IL-22 in vivo. Western blotting assay was performed to detect the regulation of the intracellular signaling pathway of IL-22.

**Principal Findings:**

We found that IL-22 suppressed the growth of A498 cells in a dose-dependent manner and inhibited the growth of A498 xenografts. We also observed that IL-22 produced a dose-dependent inhibitory effect on A498 cells that involved the induction of G2/M cell cycle arrest without cell apoptosis. Moreover, we showed that the phosphorylation of STAT1 was increased and the phosphorylation of ERK1/2 was attenuated in A498 cells exposed to IL-22. The growth inhibition of A498 cells was partially revised after IL-22 treatment as the expression of STAT1 was knocked down. And inflammatory cytokines, interferon-α and tumor necrosis factor-α (TNF-α) were barely involved in the suppression of A498 cell xenografts treated with IL-22.

**Conclusions:**

IL-22 dose-dependently suppresses RCC cell line A498 cells in vitro and induces growth inhibition of A498 cell-bearing mouse xenografts. These results suggest that the anti-RCC effects of IL-22 are at least partially mediated through regulation of STAT1 signaling pathways and G2/M cell cycle arrest, rather than by inducing apoptosis and inflammatory cytokines.

## Introduction

RCC is one of the most common malignant tumors arising in the kidney [1], [2]; chemotherapeutic agents typically have little or no impact on this type of tumor [3]–[5]. In patients with RCC, there is poor survival following the development of metastatic disease; the 5-year survival rate for these patients is less than 20% [6], [7]. Although immunotherapy with interleukin 2 (IL-2) and interferon-α (IFN-α) has been the standard treatment in patients with metastatic RCC, the response rate of patients with the disease to such treatment is only 10∼20%, and the addition of the chemotherapeutic agent 5-FU does not notably increase the survival rate [8]–[10]. Therefore, there is currently an ongoing search for new and effective cytokine therapies for RCC.

IL-22, discovered and reported by Dumoutier et al. in 2000, is a member of the IL-10 family of cytokines. IL-22 was identified as a T-cell-derived inducible factor produced by IL-9-activated murine T cells [11]. It has been found to represent an important effector molecule for activated Th1-, Th22-, Th17-, and Tc-cell subsets, natural killer (NK) and NKT cells [12]–[16]. In contrast to other cytokines, IL-22 does not mediate autocrine or paracrine functions between leukocytes, but instead serves as a mediator of communication between these cells. IL-22 may exert multiple effects on the immune system and may be involved in the acute phase response, activation of the innate immune system, induction of cell migration, inhibition of dentritic cell (DC) functions and attenuation of allergic responses [15]–[20]. Recent studies have shown that IL-22-producing T cells are more highly concentrated in lung TB granuloma than in blood and lymphoid tissues and that they contribute to anti-tuberculosis responses [12]. In addition, high systemic levels of IL-22, as well as of IL-10 and C-related Protein (CRP), in HIV-1C-infected Indian patients are associated with low viral replication *in vitro* [21]. IL-22 mediates its effects via a heterodimeric transmembrane receptor complex consisting of IL-22R and IL-10R2. It sequentially regulates several intracellular signal pathways including Janus kinase-signal transducers and activators of transcription (JAK-STAT) pathways including STAT3, Jak1 and Tyk2 [22]–[25].

Some studies support the notion that IL-22 may play different roles in different tumor cells. Although the growth of Colon 26/IL-22 tumors in syngeneic mice did not differ from that of parent tumors, survival of mice inoculated with Colon 26/IL-22 tumors was significantly prolonged compared with the survival of mice inoculated with parent tumors [26]. IL-22 inhibited the growth of human mammary adenocarcinoma EMT6 cells both *in vivo* and *in vitro* [27]. In some respects IL-22 acts synergistically with tumor necrosis factor-α, IL-1β, and IL-17. For example, IL-22 was highly expressed in non-small cell lung carcinoma, and the overexpression of IL-22 protected lung cancer cell lines from serum starvation-induced and chemotherapeutic drug-induced apoptosis; furthermore, administration of IL-22-RNAi significantly inhibited human lung tumor cell growth in BALB/c nude mice [28]. Despite these intriguing results, the functions of IL-22 are not clearly understood.

In the present study, we investigated the effects of IL-22 on human RCC cell line A498 cells *in vitro* and *in vivo* and studied the possible mechanisms underlying the anti-tumor effects of this cytokine. We found that IL-22 dose-dependently suppresses A498 cell growth and that it inhibits the growth of A498 xenografts. We also found that IL-22 induces G2/M cell cycle arrest without causing cancer cell apoptosis. In addition, we showed that the phosphorylation of STAT1 is increased and the phosphorylation of ERK1/2 is attenuated in A498 cells exposed to IL-22. The expression of tumor necrosis factor (TNF)-α and interferon (IFN)–α were not increased vigorously in A498 xenografts treated with IL-22. These results suggest that the anti-RCC effects of IL-22 are directly mediated by regulation of the STAT1 signaling pathways and G2/M cell cycle arrest rather than by induction of apoptosis or inflammatory cytokines.

## Results

### IL-22 inhibits the growth of A498 cells in vitro

**Figure 1:**
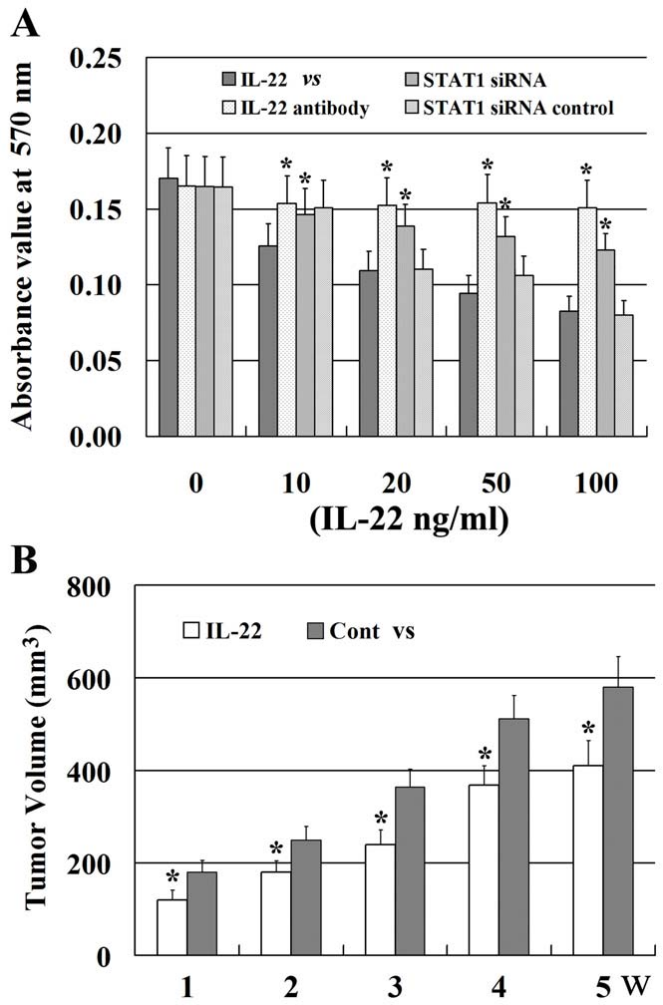
Suppression of A498 cell growth in vitro and in vivo by rhIL-22. A. IL-22 suppressed the growth of A498 cells *in vitro* in a dose-dependent manner. The growth inhibition of A498 cells exposed to IL-22 was revised by IL-22 antibody neutralization or by STAT1 specific siRNA transfection compared with the control (*P<0.05). Compared with A498 cells transfected with STAT1 siRNA, the growth inhibition of A498 cells treated with IL-22 antibody was not statistically different after IL-22 exposure (P>0.05). N = 4 and mean±SD for all groups. B. Suppressive effect of rhIL-22 on A498-cell-bearing xenografts in mice. When tumors reached 100 mm^3^, rhIL-22 was injected into the tail vein 0.5 µg /day for 7 days; control mice were injected with the same volume of PBS. Tumor size was measured with a caliper each week for five weeks; tumor volume was determined by measuring the maximal (a) and minimal (b) diameters using a caliber and calculating using the formula a×b^2^. After five weeks, mice were sacrificed under deep anesthesia and the final volume of the tumors was measured. *P<0.05 compared with control. N = 24 and mean ± SD for all groups.

We used MTT assay to investigate the effects of rhIL-22 on A498 cells and found that the final absorbance values at 570 nm were 0.1254, 0.1092, 0.0945 and 0.0825 respectively after treated with rhIL-22 at doses of 10, 20, 50 and 100 ng/ml. The corresponding inhibition rates of A498 cells were 16.4%, 27.2%, 37.0% and 45% respectively and significantly lower than the control. (**Fig. 1A**). The IC_50_ value was approximately 115 ng/ml. These results indicate that rhIL-22 dose-dependently suppresses the growth of A498 cells. Moreover, to confirm the contribution of IL-22 on growth inhibition of A498 cells, monoclonal human IL-22 antibody was used to neutralize IL-22. Interestingly, the growth inhibition of A498 cells was revised to 9.7%, 10.4%, 9.4% and 11.3% with corresponding absorbance values of 0.1535, 0.1523, 0.1540 and 0.1508 respectively (**Fig. 1A**).

### IL-22 suppresses tumor proliferation in A498 cell-bearing mice

To determine whether IL-22 has anti-tumor effects *in vivo*, 5×10^6^ A498 cells were injected (s.c.) into the neck regions of BALB/C nude mice. In the course of the experiment, we found that the average volume of tumors was about 100 mm^3^ three weeks after injection. The mice were treated with rhIL-22 (0.5 µg/day) for seven days; control mice that also received tumor cell injections were treated with PBS. Tumor diameter was measured once per week after rhIL-22 injection for five weeks. RCC xenografts were proved by the slice pathology without significantly infiltrating of leukocytes. The average volume of the tumors of rhIL-22-treated mice was significantly smaller than that of the tumors of control mice at each time point (**Fig. 1B**).

**Figure 2:**
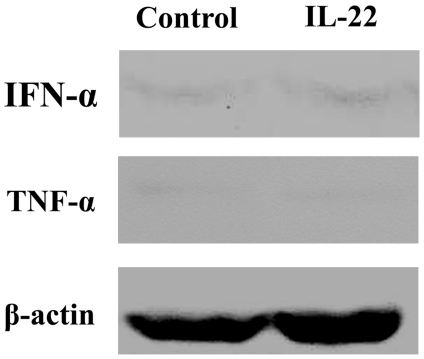
Expression of IFN-α and TNF-α in A498 xenografts after rIL-22 treatment. Western blot assay was performed to detect the expression of IFN-α and TNF-α in the A498 cell xenografts. Neither the expression of IFN-α nor TNF-α were increased significantly in A498 cell xenografts treated with rIL-22 (P>0.05 compared with control). N = 2.

To further understand the modulations in cytokines expression in A498 xenografts after rhIL-22 treatment, blotting of TNF-α and IFN-α was carried out. We found that both cytokines were barely upregulated after rhIL-22 exposure compared with control (p>0.05), indicating the suppression of A498 cell xenografts dose not involve these cytokines (**Fig. 2**).

### IL-22 induces cell cycle arrest in the G2/M phase

**Figure 3:**
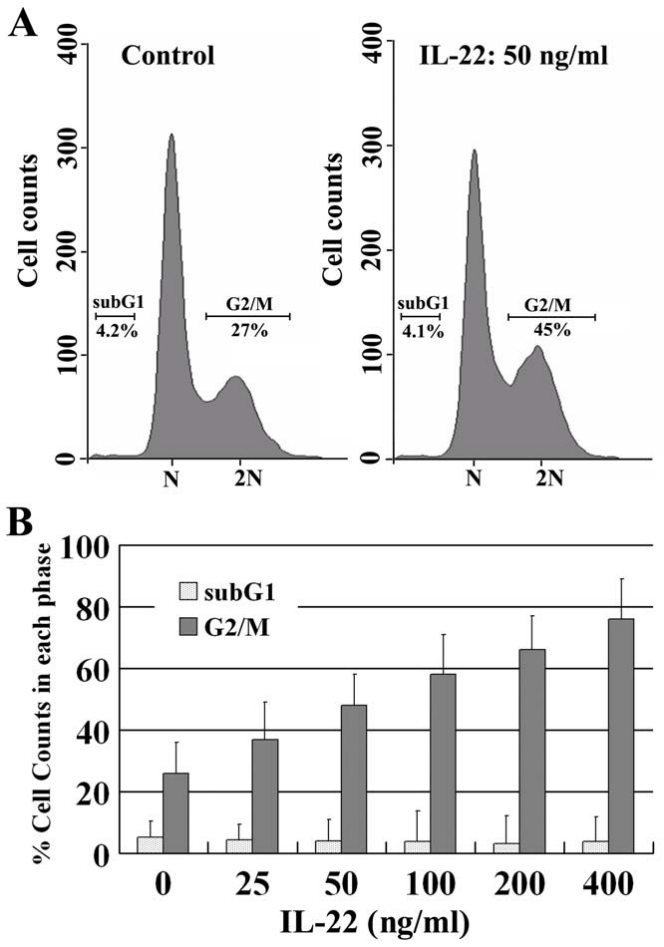
A498 cell cycle arrest in the G2/M phase after rhIL-22 exposure. A. After 50 ng/ml rhIL-22 exposure for 24 h, significantly more A498 cells accumulated in the G2/M phase of the cell cycle. B. With increasing doses of rhIL-22, more cells accumulated in the G2/M phase of the cell cycle. N = 3.

We investigated the effects of rhIL-22 on cell cycle regulation in A498 cells. FACScan analysis of propidium iodide (PI)-stained A498 cells revealed that after exposure to 50 ng/ml rhIL-22 for 24 hours, significantly more cells accumulated in the G2/M phase of the cell cycle (**Fig. 3A**). At a dose of 400 ng/ml rhIL-22, about 74% of the cells were arrested in the G2/M phase (**Fig. 3B**). These results show that the extent of cell cycle arrest is dependent on the dose of rhIL-22. Taken together the MTT results and the cell cycle arrest data, these results show that IL-22 inhibits A498 tumor cell proliferation both *in vitro* and *in vivo*

### IL-22 receptor detection by western blot assay

**Figure 4:**
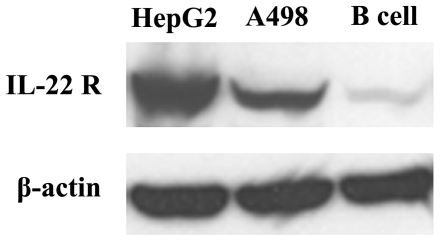
Expression of IL-22R on the surface of cells by western blot assay. Western blot assay was performed to detect the expression of IL-22R on the surface of cells using mouse IL-22 Rα1 antibody. The results showed a measurable expression of IL-22R on the surface of A498 cells compared with that of HepG2 cells and human B cells, which served as positive control and negative control respectively. N = 3.

IL-22R expression has been found in many cancer cells [22]–[25]. Concerning the growth inhibition of A498 cells both *in vivo* and *in vitro* after IL-22 exposure, we reasoned that IL-22R might be expressed and might play a functional role in A498 cells. Therefore, we studied the expression of the IL-22R and functional consequences of IL-22 exposure in A498 cells. To examine the expression of IL-22R in A498 cells, we performed a western blot assay using mouse IL-22 Rα1 antibody, which displayed a measurable IL-22R expression in A498 cells. We also selected HepG2 cells and human B cells served as positive control and negative control cells of IL-22R respectively, which demonstrates a selective expression of IL-22R in different cells (**Fig. 4**).

### IL-22 regulates the STAT1 and ERK1/2 signaling pathways

**Figure 5:**
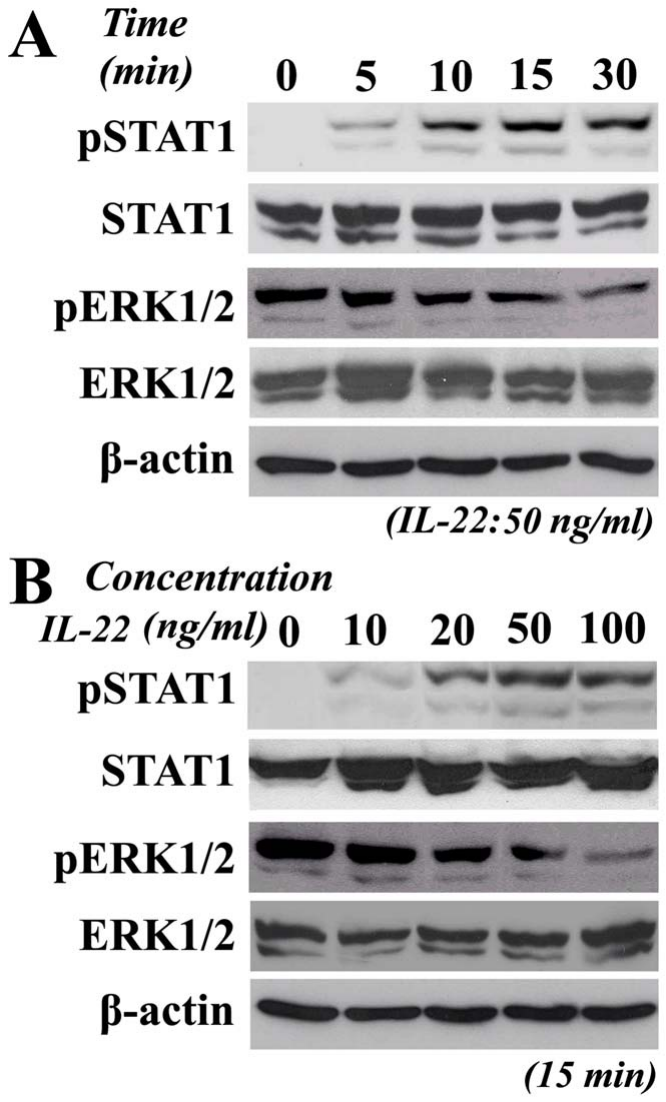
The regulation of STAT1 and ERK1/2 pathways by rhIL-22. A. p-STAT1 and p-ERK1/2 levels after 50 ng/ml rIL-22 exposure. B. p-STAT1 and p-ERK1/2 levels 15 minutes after administration of various doses of rhIL-22. N = 3.

We investigated the downstream intracellular signal mediators of IL-22 action in A498 cells. Western blotting assays showed that STAT1 was phosphorylated from the 5^th^ minute after exposure of the cells to rhIL-22 and that the phosphorylation of STAT1 (p-STAT1) reached its peak level 30 minutes after rhIL-22 exposure. In contrast to STAT1, ERK1/2 was dephosphorylated in response to rhIL-22; this effect was maximal 30 min after rhIL-22 exposure (**Fig. 5A**). Both the phosphorylation of STAT1 and the dephosphorylation of ERK1/2 were amplified with increasing doses of rhIL-22 (**Fig. 5B**). In this procedure, the concentration of STAT1 in A498 cells was stable regardless of extension the rhIL-22 exposure time. The results demonstrate that rhIL-22 activates STAT1 and inhibits ERK1/2 pathways in A498 cells in a time- and dose-dependent manner and that the biological functions of the receptors for these pathways show time-dependent STAT1 activation and ERK1/2 deactivation evoked by rhIL-22. The output of STAT1 protein in these cells treated with IL-22 is not significantly increased indicating that STAT1 protein is not extra synthesized in gene level during this procedure.

**Figure 6:**
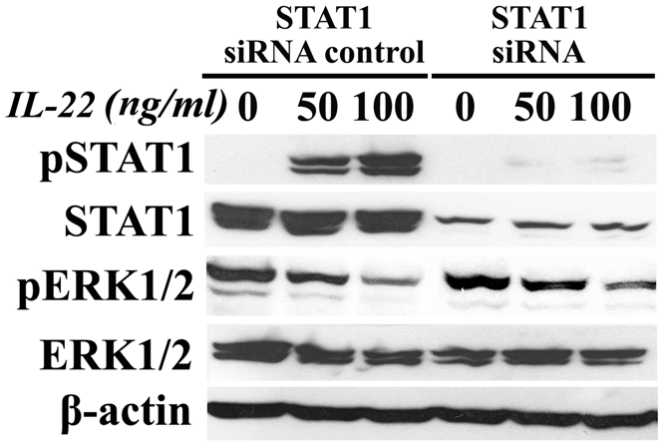
STAT1 and ERK1/2 pathways in A498 cells transfected with STAT1 siRNA by western blot assay. The expression of p-STAT1 decreased by 87.2%±3.6% in STAT1 siRNA transfected cells at the 30^th^ minute after IL-22 treatment and maintained in a lower level compared with that of negative control siRNA (P<0.05). N = 2.

To confirm that p-STAT1 has an anti-RCC effect on A498 cells, the expression of STAT1 was knocked down by STAT1 specific siRNA transfection. It revealed that p-STAT1 was maintained in a lower level in transfected cells after IL-22 treatment (87.2%±3.6% decrease relative to control) compared with that of negative control siRNA (**Fig. 6**) and significant lower growth inhibition rates of these cells (11.3%, 15.7%, 17.2% and 21.4%) were observed when treated with 10, 20, 50 and 100 ng/ml of rhIL-22compared with that of A498 cells without siRNA transfection. Although the phosphorylation of ERK1/2 was still attenuated during this procedure, the absolute contribution of STAT1 pathway to the growth inhibition of A498 cells was observed via the limited revision of these cells' growth inhibition by dephosphorylation ERK1/2 alone when STAT1 was knocked down. Moreover, the cells transfected with negative control siRNA had a comparative growth inhibition rate after exposure to rhIL-22 with that of A498 cells (**Fig. 1A**).

### Lack of apoptosis of A498 cells after IL-22 exposure

**Figure 7:**
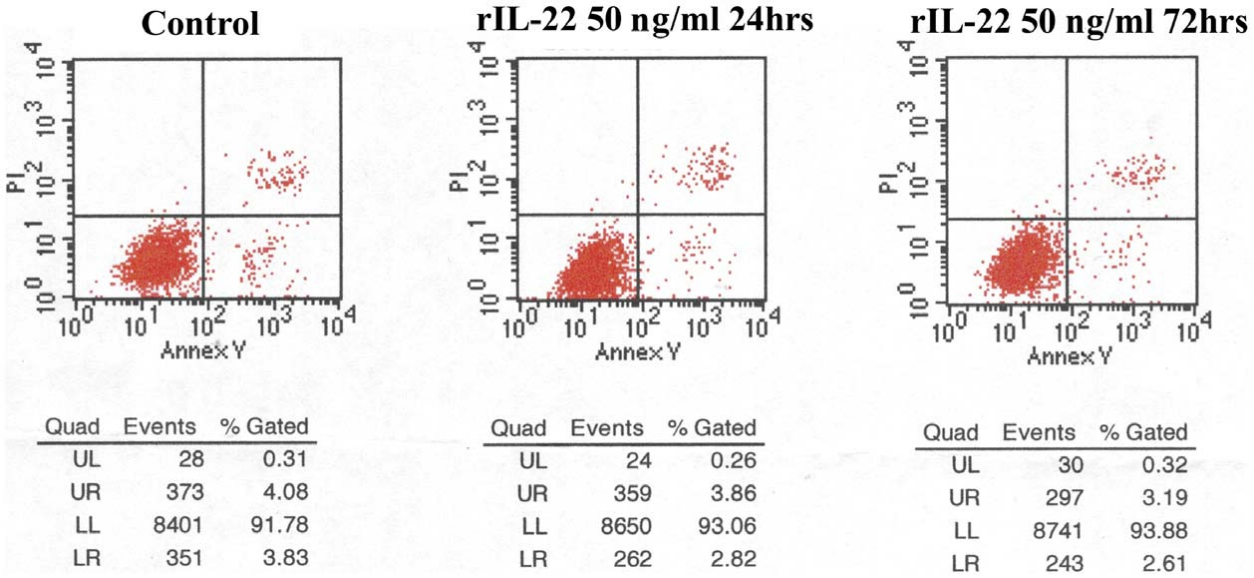
Lack of apoptosis in A498 cells after rhIL-22 treatment. A. Treatment of A498 cells with 50 ng/ml rhIL-22 for 24 h did not induce apoptosis as measured by annexin V or PI-positive cells. B. Treatment of A498 cells with 50 ng/ml rhIL-22 for 72 h did not induce apoptosis. N = 3.

We next determined whether inhibition of tumor growth by IL-22 involves the induction of apoptosis. We used FACScan to measure annexin V binding in A498 cells treated with doses of rhIL-22 ranging from 0 to 400 ng/ml for times ranging from 24 to 72 hrs. The analysis showed that the fraction of annexin-V-positive/ PI-positive cells did not change significantly after rhIL-22 treatment, even at a dose of 400 ng/ml rhIL-22 and an exposure time of 72 hrs (**Fig. 7**). Taken together with the results of the FACScan analysis of the PI-stained cells, these data suggest that IL-22 treatment induces cell cycle arrest in the G2/M phase but does not induce apoptosis in A498 cells.

## Discussion

In the current study, we investigated the effects of IL-22, a member of the IL-10 cytokine family, on human RCC cell line A498 cells *in vitro* and *in vivo* and studied the possible mechanisms underlying the anti-RCC tumor effects of the cytokine. First, we found that IL-22 receptors are expressed in A498 cells and that IL-22 dose-dependently suppresses A498 cell growth and inhibits A498 xenograft growth. Second, we observed that IL-22 induced G2/M cell cycle arrest occurs without cell apoptosis. Moreover, we showed that the phosphorylation of STAT1 is increased and the phosphorylation of ERK1/2 is attenuated in A498 cells exposed to IL-22. Indeed, the growth inhibition of A498 cells exposed to IL-22 was partially revised to a relatively lower level after STAT1 was knocked down, indicating that STAT1 pathway plays a more important role in the growth inhibition of A498 cells exposed to IL-22 than ERK1/2 pathway does. We also showed that IL-22 did not enhance the anti-tumor immunity effectively *in vivo* by increasing the expression of IFN-α and TNF-α in A498 cell xenografts. These results suggest that the anti-A498 tumor effects of IL-22 are directly mediated by up-regulation of STAT1 and G2/M cell cycle arrest rather than by inducing apoptosis and inflammatory cytokines.

Epidemiological studies have shown that the 5-year survival rate of advanced RCC patients is only 9.5% [2]. RCC is resistant to chemotherapy, and chemotherapeutic agents alone achieve a clinic response rate of less than 10%. Treatment regimens involving the administration of IFN-α have been used in patients with RCC with therapeutic response rates around 4–33% [29]. The low response rate, toxicity associated with high-doses regimens and low long-term survival rate of immunotherapy emphasize the necessity of finding a new agent to deal with RCC; the current lack of therapeutic agents for RCC that fulfill the basic and necessary criteria for clinical treatment demand the development of more effective drugs to combat this disease.

IL-22, which in humans is mainly produced by Th22-, Th1- and Th17-cells, is strongly involved in immune regulation and inflammatory responses [19], [27], [28], [30]. IL-22 plays an important role in inflammatory processes through up-regulation of acute phase reactions and of pancreatitis-associated protein [14], [15], [22], [23], [31]. However, there is some debate about the effects of IL-22 on tumors. We suppose that IL-22 may play different roles in different tumors and that discrepancies in experimental results may be due to different cell types and diseases. The results of the present study support the idea that IL-22 suppresses the growth of A498 cells both *in vivo* and *in vitro*.

The functional IL-22 receptor complex consists of two receptor chains, IL-22R and IL-10R2. IL-22R2 is broadly expressed in various tissues [24], [32], and the expression of the IL-22R chain determines whether a cell will be a target of IL-22. In our study, we detected measurable expression of IL-22R in A498 cells and showed that IL-22 suppressed the growth of RCC cells in a dose-dependent manner. Similar effects were obtained in A498 xenograft tumors.

Although IL-22 is produced by immune cells, unlike other cytokines it does not affect immune cells directly but instead regulates the functions of their target cells, increasing the innate immunity of tissue cells, protecting tissues from damage and enhancing their regeneration. The signal transducer and activator of transcription 1 (STAT1) plays a critical role in carcinogenesis by mediating various biological responses and has been implicated as a tumor suppressor [33]. Both carcinogen-induced and transplanted tumors are increased in STAT1 knockout mice cpmpared with wild-type control mice [34], [35]. Zhu demonstrated that SNPs in the STAT1 gene (homozygotes of the minor alleles at SNP rs867637, rs3771300, or rs2280235) are associated with a risk of developing hepatocellular carcinoma in Chinese people [36]. While, Specific inhibition of ERK1/2 phosphorylation by a variety of natural and synthetic compounds has been shown to be effective in anticancer strategy in the treatment of breast cancer [37], [38].To better understand the molecular mechanisms that underlie the anti-RCC tumor effects of IL-22, we assessed the phosphorylation of STAT1 and ERK1/2 in A498 cells after rhIL-22 treatment. Our western blotting assays showed that p-STAT1 was founded in A498 cells at the 5^th^ minute and reached its peak level 30 minutes after rhIL-22 exposure. While ERK1/2 was dephosphorylated in response to rhIL-22; this effect was maximal 30 min after rhIL-22 exposure. It suggests that rhIL-22 activates STAT1 and inhibits ERK1/2 pathways in A498 cells in a time- and dose-dependent manner and that the biological functions of the receptors for these pathways show time-dependent STAT1 activation and ERK1/2 deactivation evoked by rhIL-22. Concerning the concentration of STAT1 protein in A498 cells after IL-22 exposure was stable independent on the vary dose of IL-22 and longer exposure time, we believe that IL-22 induce STAT1 pathway activating directly rather than act on gene level. Hence, the STAT1-ERK1/2 pathway participates, at least in part, in the inhibition of A498 cell growth that occurs after IL-22 binding to and activation of IL-22R. To further understand the role of STAT1-ERK1/2 pathway in the growth inhibition of A498 cells exposed to IL-22, the expression of STAT1 was deleted in A498 cells by STAT1 specific siRNA. The phosphorylation STAT1 was decreased by 87.2% after treated with IL-22 compared with the controls and independent to dephosphorylated ERK1/2 pathway.The results show that the growth inhibition of A498 cells with STAT1 deletion was only partially revised in these cells treated with IL-22 compared with the controls (p>0.05), which indicates that the activation of STAT1 pathway is the major mechanism involved in the growth inhibition of A498 cells rather than dephosphorylation of ERK1/2. Dysfunctional STAT1 may contribute to cancer development and progression, while at the same time the ERK1/2 pathway regulates a common set of cell death regulators such as BCL-2, BCL-XL and BIM, indicating that it plays a role in cell cycle control [39]–[41]. ERK1/2 inhibitors would therefore be expected to function as anti-tumor agents [37], [38]. Concerning the lack of contribution of ERK1/2 pathway to the growth inhibition of A498 cells exposed to IL-22, it can be hardly concluded that IL-22 could serve as a reasonable ERK1/2 inhibitor for A498 cells. (Figure S1: a schematic diagram for STAT1-ERK1/2 pathway work in this procedure.)

We propose that the STAT1 pathway is activated in A498 cells after IL-22 treatment. Because activation of the STAT1 pathway usually leads to apoptosis, we further investigated whether IL-22 suppresses the proliferation of RCC cells by inducing apoptosis or cell-cycle arrest. Our results showed that IL-22 did not induce apoptosis in A498 cells but instead was associated in a dose-dependent manner with cell cycle arrest in the G2/M phase.

Our results also showed a tumor growth inhibition effect *in vivo* when exposure to IL-22. After treated with IL-22, the A498 xenografts were significantly smaller than that of control mice. In this study, we used BALB/c nu mice as tumor-bearing host and the tumors were confirmed in pathology. To further understand the function of inflammatory cytokines in this procedure, IFN-α and TNF-α were analyzed by western blotting assay. It was found that the levels of TNF-α and IFN-α remained unchanged in A498 xenografts after IL-22 treatment compared with that of controls (p>0.05). TNF-α was originally described as an endotoxin-induced, macrophage-derived protein with different effects depending on the tumor type to which it is administered [42]. The results of minimal changes of TNF-α and IFN-α in A498 xenografts tissue after IL-22 exposure suggest minimal immunocytes recruitment in this procedure. The tumor infiltration leukocytes were rare in both IL-22 treatment tumors and controls probably due to the adoption of athymic BALB/c nu mice as the xenograft host. Hence, we reasoned that IL-22 act as an immediate anti-A498 cell actor in this procedure rather than a promoter cellular immunity. However, we do not deny that other unknown anti-A498 cells mechanisms take part in this procedure and further research in this field is necessary.

Several studies focusing on the effects of IL-22 on cancer cells have shown similar results. IL-22 could significantly prolong the survival of mice bearing Colon 26 cells, though it did not inhibit the growth of colon cancer cells [26]. IL-22 could reduce the growth of mammary adenocarcinoma (EMT6) cells; this growth reduction was associated with the inhibition of ERK1/2 and Akt phosphorylation and the induction of G2/M phase cell cycle arrest [27]. Interestingly, in contrast to its protective role in some cells, IL-22 seems to serve as a tumor promoter in other human tumor cells, likely as a result of its regulation of different intracellular signaling pathways in different cell types. The growth of HepG2 human hepatocellular carcinoma cells was promoted by IL-22 through the activation of STAT3, ERK1/2 and the induction of antiapoptotic proteins [32]. IL-22 also protected lung cancer cell lines from serum-starvation-induced and chemotherapeutic drug-induced apoptosis by activation of STAT3 and its downstream antiapoptotic proteins and inhibition extracellular signal-regulated kinase 1/2 [28]. Based on these results, we presume that the different observed effects of IL-22 depend on its activation of different cell signaling pathways in different tissue types. Recently, the association between IL-22 genetic polymorphisms and the risk of colon cancer was investigated; it shows that one haplotype containing the rs1179251 G allele with the incidence of 21.03%, which enhance the IL-22 exposure, gave an estimated 52% increase in risk of colon cancer for individuals [30]. However, the SNP (rs1179251) is a non-coding (intronic) SNP. Therefore, it is uncertain whether IL-22 plays the role of a potential promoter in colon cancer [30]. Due to the controversial biological effects of IL-22 in tumor cells, it is necessary to carry out further research on this topic.

In summary, we have demonstrated that IL-22 inhibits the growth of A498 cells (RCC) both *in vivo* and *in vitro*, at least partially by activating the STAT1 pathway directly rather than acting on gene level in these cells. The anti-A498 cells effects of IL-22 were associated with cell cycle arrest in the G2/M phase. Apoptosis and immunocytes recruitment were not found to be involved in this process. We suggest that IL-22 may be an effective agent for human RCC A498 cells growth inhibition; further, the STAT1 pathway could be an interesting target for improving patient responses in RCC. The data presented here support this conjecture, although additional experimental on other RCC cell lines and clinical evidence will be required to advance this hypothesis.

## Materials and Methods

### Cell culture

The human A-498RCC cell line was purchased from Cell Bank of China Union Medical University (Beijing, China) and cultured in 5% CO_2_ humidified atmosphere at 37°C in complete RPMI-1640 medium (GIBCO, UK) supplemented with 25 mM HEPES, 2 mM L-glutamine, 1% non-essential amino acids, 100 µg/ml penicillin, 100 µg/ml streptomycin and 10% heat-inactivated fetal bovine serum (FBS).

### Growth inhibition assay

Exponentially growing A498 cells were seeded at 1,000 cells/well in a 96-well microculture plate. Multi-dosage of recombinant human interleukin 22 (rhIL-22, ADL), 10 ng, 20 ng, 50 ng and 100 ng respectively, were added after cell adhesion for 4 hrs followed by continuous incubation for 3 days. Cells treated with PBS served as controls. 20 µl MTT (5 mg/ml) was added to each well and the plate was incubated at 37°C for another 4 h, after which 10% HCl-SDS (100 µl) was added to each well. The plate was kept at 4°C overnight. Cells were then harvested and cell proliferation was determined using a 96-well plate reader to record the absorbance at 570 nm. The percentage inhibition of A498 cells was calculated using the following formula: % inhibition = (absorbance of control well−absorbance of IL-22 well)/absorbance of control well×100%.

Monoclonal human IL-22 antibody (300 µg/well, R&D) was used to confirm the contribution of IL-22 to A498 cells growth. The percentage inhibition of A498 cells treated with different doses of rhIL-22 was re-calculated with the similar procedures.

#### Growth inhibition activity of IL-22 on A498 xenografts

BALB/C nude mice provided by SLAC Laboratory Animal (Shanghai, China), were studied after approval from the Medical ethics committee of Beijing Friendship Hospital, Capital Medical University. Three- to four-week-old mice were maintained in high-efficiency particulate air-filtered cages in a pathogen-free facility. A498 cells were washed once and resuspended in serum-free medium. 5×10^6^ cells in matrigel (BD Biosciences, San Jose, CA) were injected into the neck region; mice were examined the day after injection. When the tumor size reached 100 mm^3^, rhIL-22 was injected into the tail vein (0.5 µg /day for 7 days). Control mice were injected with the same volume of PBS. Tumor size was measured with a caliper each week for 5 weeks; the tumor volume was determined by measuring the maximal (a) and minimal (b) diameters using a caliber and calculated by using the formula a×b^2^. After five weeks, mice were sacrificed under deep anesthesia and the final volumes of the tumors were measured.

### Flow cytometric analysis of cell cycle status

A498 cells (1×10^4^) were seeded on 10-cm dishes and incubated for 24 hrs in serum-free medium. Cells were then treated with 25, 50, 100, 200 or 400 ng/ml of rhIL-22 for 24 hrs or with PBS as control. The cells were then collected, fixed with 70% ethanol at −20°C overnight, washed 3 times with PBS and incubated at 37°C for 30 min in 7-amino-actinomycin D (7-AAD) staining solution (BD Biosciences Pharmingen). Cell counts in each phase of the cell cycle were estimated using a FACSCalibur (Beckton Dickinson) and Cellquest 3.0 software.

### Western blotting analysis of IL-22R on the surface of cells

A498 cells (1×10^6^) were seeded into 6-well plates and cultured for 6 hours in serum-free medium. Total cell lysates were prepared on ice in a buffer containing 50 mM Tris-HCl (pH 8.0), 150 mM NaCl, 1% Nonidet P-40, 0.5% deoxycholic acid, 0.1% SDS and 1 mM EDTA. Proteins were isolated by 10% SDS-PAGE and the concentration of protein was assessed using the Bradford dye-binding protein assay (Bio-Rad, Richmond, CA, USA), and SDS-polyacrylamide gel electrophoresis was performed. Mouse IL-22 Rα1 antibody was purchased from R&D system. Anti- β-actin monoclonal antibody (Abcam, Cambridge, UK) was used as an internal loading control. The immune complexes were detected using the ECL plus Western Blotting Detection System (Amersham, Aylesbury, UK).

Hepatoma cell line HepG2, purchased from Cell Bank of China Union Medical University (Beijing, China) and cultured as A498 cells, was served as positive control of IL-22R. As for negative control, we selected human B cells purified from human spleen. The splenic tissue was gathered from a patient with informed consent who was performed splenectomy due to splenic rupture. The procedure of B cells purification was followed the statement of LeÂcart [43]. The purified B cells populations were confirmed CD19+ >98% by flow cytometry assay. The detection procedure of IL-22R on the surface of these cells was similar as showed above.

### Western blotting analysis of p-STAT1 and p-ERK1/2 after IL-22 exposure

A498 cells (1×10^6^) were seeded onto 6-well plates and cultured for 6 hours in serum-free medium. Cells were then treated with 10 ng/ml, 20 ng/ml, 50 ng/ml and 100 ng/ml rhIL-22 for 5 min, 10 min, 15 min, 20 min, and 30 min respectively or with PBS as control. Total cell lysates were prepared in a buffer containing 50 mM Tris-HCl (pH 8.0), 150 mM NaCl, 1% Nonidet P-40, 0.5% deoxycholic acid, 0.1% SDS and 1 mM EDTA on ice. Samples were sonicated for 30 seconds and boiled at 95°C for 5 min. Proteins were isolated by 10% SDS-PAGE and the concentration of protein was assessed using the Bradford dye-binding protein assay (Bio-Rad, Richmond, CA, USA), and SDS-polyacrylamide gel electrophoresis was performed. phospho-STAT1 (Tyr701)/STAT1 (9H2) antibody and phspho-ERK1/2 (Thr202/Tyr204)/MAPK (Erk1/2) antibody were purchased from Cell Signaling. Anti- β-actin monoclonal antibody (Abcam, Cambridge, UK) was used as an internal loading control. The immune complexes were detected using the ECL plus Western Blotting Detection System (Amersham, Aylesbury, UK).

### STAT1 small interfering RNA

STAT1 small interfering RNA (siRNA) sequences were used to removal the contribution of STAT1 on cells growth. STAT1 siRNA sense sequence r(CACGAGACCAAUGGUGUGG)d(TT) or STAT1 target siRNA anti-sense sequence r(CCACACCAUUGGUCUCGUG) d(TT) ( all from Qiagen) was used to knock down STAT1 expression (DNA target sequence: AAC ACG AGA CCA ATG GTG TGG; GenBank accession no. NM-139266, nucleotides 893–913). A scrambled sequence was used as a negative control sequence. The STAT1 siRNA transfection was performed following the Qiagen TransMessenger transfection protocol (Qiagen Inc, Valencia, CA). Cells were transfected with siRNA and incubated for 48 hrs. The same dose of IL-22 treated siRNA transfected A-498 cells as above shows and proteins expression such as STAT1, p-STAT1 were assessed by Western blot assay.

#### Growth inhibition activity of IL-22 on A498 cells transfected with STAT1 siRNA

A498 cells transfected with STAT1 siRNA was used to confirm the role of STAT1 in cell proliferation after treated with IL-22. The 1,000 cells/well of STAT1 siRNA A498 cells were exposure to IL-22 with the same concentration gradient. The growth inhibition assay was performed as A498 cells'.

### Flow cytometric analysis of apoptosis

A498 cells (1×10^6^ /well) were seeded onto 6-well plates. After allowing 6 hours for the cells to adhere, the cells were treated with rhIL-22 (with the dose of 25, 50, 100, 200 and 400 ng/ml respectively) for 24 or 72 hrs respectively. The cells were then trypsinized, washed with binding buffer, and incubated with FITC-labeled annexin V antibody for about 20 min at 37°C in the dark. The cells were resuspended and 1% FCS and 10 µl of propidium iodide (PI)^3^ solution (1 mg/ml) were added. Flow cytometry was performed in a FACScan with a single cell gate (BD Biosciences). The dot plots in quadrants quantified the percentage of cells.

#### Western blotting analysis of IFN-α and TNF-α in A498 xenografts

The tumor tissues were harvested after the mice were sacrificed and the samples were immediately frozen and crushed into powder in liquid nitrogen. Ice-cold lysis buffer (T-PER, Pierce) with protease inhibitor (Complete Mini, Roche) was added and then kept on ice for 30 min. Proteins were isolated by 10% SDS-PAGE and the protein concentration was estimated by Bradford dye-binding protein assay (Bio-Rad, Richmond, CA, USA) in accordance with the manufacturer's protocol.

The protein (30 µg) were dissolved in 10–12% polyacrylamide-SDS gels and transferred onto a nitrocellulose membrane. The membrane was immersed in blocking buffer (5% non-fat dry milk/1% Tween 20 in 20 mM TBS, pH 7.5) for 1.5 h at room temperature and incubated with the appropriate primary antibodies, mouse monoclonal anti-TNF-α (1∶ 50) and IFN-α (1∶100) (Santa Cruz Biotechnology, Santa Cruz, CA, USA) in blocking buffer overnight at 4°C, followed by incubation with complementary secondary antibody. Proteins were detected by the ECL plus Western Blotting Detection System (Amersham, Aylesbury, UK).

#### Statistical analysis

All experiments were carried out in triplicate, and continuous variables are expressed as mean ± SD. For statistical analysis, continuous variables that met the criteria for a normal distribution were determined using a two-tailed Student's *t*-test. Statistical analysis of the results of the flow cytometry experiments was performed using the Fisher's exact test and two-sided tests. The χ^2^ test was used for numeration data. Significance for all tests was established at P<0.05.

## Supporting Information

Figure S1 **Schematic diagram for the mechanism of IL-22 effect on A498 cells.** After IL-22 bind with IL-22R and IL-10R2, the IL-22 receipt compound is formed and the activation of STAT1 and deactivation of ERK1/2 pathway were followed, which results the growth inhibition of A498 cells.
(TIF)
