# Supplementary figures and images for: Interleukin-22 Suppresses the Growth of A498 Renal Cell Carcinoma Cells via Regulation of STAT1 Pathway
Source: PLoS One. 2011 May 23;6(5):e20382. doi: 10.1371/journal.pone.0020382 (PMC3100322; doi:10.1371/journal.pone.0020382)

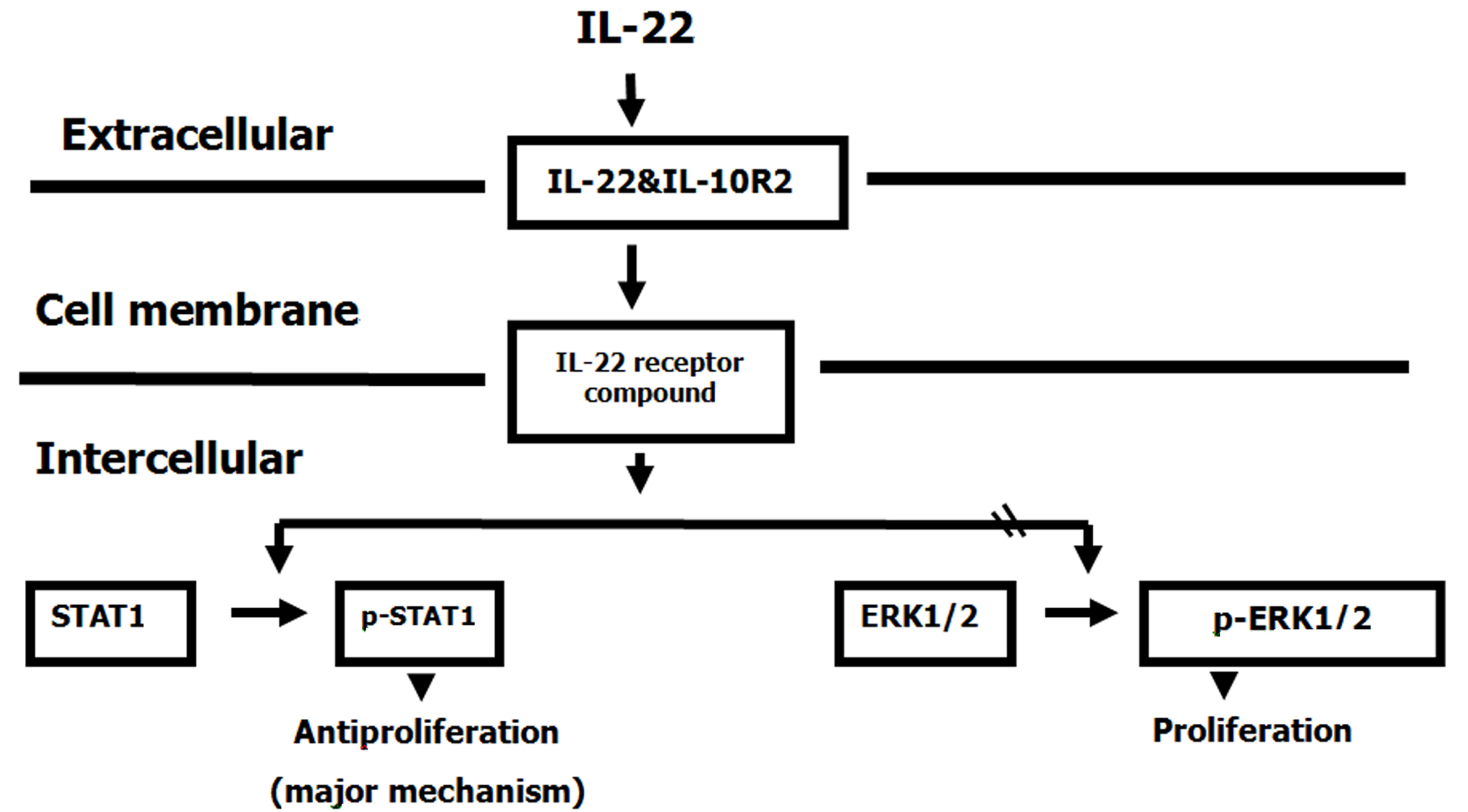

Supplement: Figure S1 — Schematic diagram for the mechanism of IL-22 effect on A498 cells. After IL-22 bind with IL-22R and IL-10R2, the IL-22 receipt compound is formed and the activation of STAT1 and deactivation of ERK1/2 pathway were followed, which results the growth inhibition of A498 cells. (TIF) [file pone.0020382.s001.tif]
